# Broiler Chicken Behavior and Activity Are Affected by Novel Flooring Treatments

**DOI:** 10.3390/ani11102841

**Published:** 2021-09-29

**Authors:** Leonie Jacobs, Shawnna Melick, Nathan Freeman, An Garmyn, Frank A. M. Tuyttens

**Affiliations:** 1 Department of Animal and Poultry Sciences, Virginia Polytechnic Institute and State University, 175 West Campus Drive, Blacksburg, VA 24061, USA; shawnna5@vt.edu (S.M.); nathanf1@vt.edu (N.F.); 2 Faculty of Veterinary Medicine, Ghent University, 9820 Merelbeke, Belgium; An.Garmyn@UGent.be (A.G.); frank.tuyttens@ilvo.vlaanderen.be (F.A.M.T.); 3 Flanders Research Institute for Agriculture, Fisheries and Food (ILVO), 9090 Melle, Belgium

**Keywords:** animal welfare, animal behavior, normal behavior, flooring, meat birds, ethology

## Abstract

**Simple Summary:**

Broiler chickens should be able to express highly motivated behaviors, such as foraging and dustbathing. Health status and housing conditions impact the expression of these behaviors. This study compared the impact of novel flooring treatments on broiler chicken behavioral repertoire. We found that broilers’ behavior was impacted by novel flooring treatments at 5 and 6 weeks of age. Differences were found in prevalences of drinking, foraging, preening, locomoting, and in generally being active. Generally, broilers with access to clean friable litter spent more time drinking, foraging, locomoting, preening and being active compared to when housed with a partially slatted floor and/or a disinfectant mat. Thus, access to clean, regularly replaced litter is beneficial for broiler chicken welfare, especially for their ability to perform normal behaviors.

**Abstract:**

The objective was to determine broiler chicken behavioral differences in response to novel flooring treatments. Broilers (*n* = 182) were housed in 14 pens (a random subset from a larger-scale study including 42 pens), with 13 birds/pen. One of seven flooring treatments were randomly allocated to 14 pens (2 pens per treatment). The flooring treatments (provided from day 1 {1} or day 29 {29}) included regularly replaced shavings (POS), a mat with 1% povidone-iodine solution (MAT), and the iodine mat placed on a partially slatted floor (SLAT). In addition, a negative control treatment was included with birds kept on used litter from day 1 (NEG). Behavior was recorded in weeks 1, 2, 5, and 6. In week 5, treatments affected the behavioral repertoire (*p* ≤ 0.035). Birds in POS-1 showed more locomoting, preening and activity overall compared to MAT and/or SLAT treatments. Birds in POS-29 showed more drinking, foraging, preening and overall activity than birds in MAT and/or SLAT treatments. In week 6, birds in the POS-1 treatment spent more time foraging compared to birds in all MAT and SLAT treatments (*p* ≤ 0.030). In addition, birds in the POS-1 treatment spent more time preening than birds in the MAT-1 treatment (*p* = 0.046). Our results indicate that access to partially slatted flooring and/or disinfectant mats does not benefit broiler chicken welfare in terms of their ability to express highly motivated behaviors. Access to clean, regularly replaced litter is beneficial for broiler chicken welfare in terms of their ability to express their normal behavioral repertoire.

## 1. Introduction

To ensure good animal welfare it is important to allow animals the ability to express highly motivated behaviors [1,2]. For broiler chickens, species-specific, highly motivated behaviors include foraging (or scratching) and dustbathing [2,3,4]. Broiler chickens were observed spending approximately 1–10% of their time foraging [5,6] and between 0.2 and 3.7% of their time dustbathing when housed on a range of litter types [6]. These behaviors and behaviors such as preening, stretching and play are generally considered to be positive indicators for animal welfare as they are deemed to be indicative of positive affective states [7,8,9]. Inducing positive affective states is considered a key component to ensure good animal welfare [10].

Previous work has shown that accessible resources such as a substrate can impact the expression of these behaviors. For instance, access to sand resulted in more frequent dustbathing compared to pine wood shavings, rice hulls, or a paper bedding product [3]. Broilers showed more foraging when housed with access to maize roughage compared to the control group, yet access to straw bales did not impact foraging behavior [11]. More play, ground scratching and ground pecking was observed when broilers had access to enrichments (peat, hay bales and elevated platforms) compared to a control [12]. Broilers housed on partially or fully slatted floors grew faster than broilers housed on litter, and this was attributed to birds on slatted floors directing their foraging behavior to the feeder, rather than the litter [13]. However, direct observations of behavior were not performed, thus the impact of slatted flooring on broilers’ behavioral repertoire was not investigated. Other studies did not find an impact of accessible resources on foraging [14] or play [15,16].

Flooring substrate can also impact broilers’ physical condition. Contact dermatitis on feet, hocks, or breast due to prolonged contact with moisture and irritants in litter is a prevalent health and welfare concern in broiler chickens [17,18,19,20,21,22]. In turn, these skin conditions can affect the birds’ behavioral repertoire due to discomfort and pain [23,24]. Birds with contact dermatitis in a commercial setting are generally not treated to allow healing, thus lesions will likely worsen over time. We propose to investigate topical application of an antiseptic whether or not combined with a slatted floor to reduce time spent in contact with litter as potential flock-level approaches to prevent or remedy contact dermatitis. Early (day 1) or late (day 29) flock-level treatment approaches may prove beneficial to reduce dermatitis prevalence and severity, yet these treatments could affect the birds’ ability to perform highly motivated behaviors. We previously determined the impact of novel pen-level preventative and remedial approaches on contact dermatitis (including footpad dermatitis), cleanliness, gait and body weight [25]. Those results demonstrated that flooring treatments and timing of treatments affected contact dermatitis severity, with access to regularly replaced clean litter showing the lowest prevalence and severity of the welfare issues [25]. With our previous work indicating worsened footpad dermatitis when birds had access to partially slatted floors and/or mats [25], it is likely the behavioral repertoire would be impacted. Therefore, the objective of this study was to assess behavioral differences in response to novel flooring treatments at the flock level, provided at different ages (starting on day 1 or day 29 of age). We hypothesized that flooring treatments would affect the behavioral repertoire, with more highly motivated behaviors (foraging and dustbathing) shown when birds had access to clean, regularly replaced pine shavings, and a potential detrimental effect of slatted flooring and mats on the birds’ ability to show highly motivated behaviors.

## 2. Materials and Methods

This experiment was carried out between March and May 2019 and was approved by the Institutional Animal Care and Use Committee (IACUC) of Virginia Tech (protocol 18-246). For the behavioral data reported in this manuscript, a random selection of birds (182 out of 546) and pens (14 out of 42) were used from those reported in [25].

For this study, 182 commercial strain (Ross × Hubbard) male broiler chicks were housed at the poultry facility of Virginia Tech from day 1 until day 49 of age, with 13 birds per pen (1.25 m^2^). Upon arrival at the facility, birds were randomly allocated to 1 of 7 treatment groups (3 × 2 factorial + 1 industry control group). Pens contained pine shavings, a drinker line with three nipples, a feeder, and in the first week, a heat lamp and a feed flat with feed. After the first week lighting was provided for 18 h, followed by 6 h of uninterrupted darkness. Feed was provided ad libitum following commercial standards with a starter, grower and finisher phase. More details on housing conditions were reported in [25].

The experiment consisted of an incomplete factorial design, with four flooring treatments and two timing treatments. The flooring treatments included a negative control (NEG), a positive control (POS), and two novel flooring treatments with disinfectant mats containing a povidone-iodine solution (MAT and SLAT). The two timing treatments consisted of access to the flooring treatments from day 1 of age or day 29 of age onwards. Pens that received the flooring treatments from day 29 of age were kept under identical conditions as the negative control up until day 29. Treatments were randomly allocated over blocks, resulting in 14 pens and 7 treatment groups (2 replicates per treatment).

**Figure 1:**
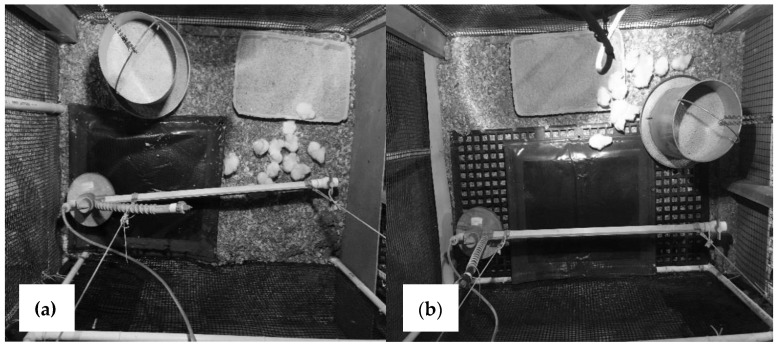
Top view photos of pen-based flooring treatments, with (**a**) a mat filled with 1% povidone-iodine solution (MAT) and (**b**) the iodine mat placed on a slatted floor (SLAT). The pens contained a hanging drinker line, a metal feeder, used litter, a disinfection mat, and for the SLAT treatment, a plastic slatted floor. In the first week, a feed flat with feed was provided.

Flooring treatments (see [25] for more details) consisted of the following:

1. The NEG flooring treatment consisted of pens with used litter (19.1% moisture content as measured prior to bird placement) that was collected from a previous broiler flock, to model an industry standard in the United States and other countries [26,27]. Litter was collected from experimental pens in the same facility and piled in the center hallway. The used litter was mixed manually and returned to the pens the next day to ensure an equal distribution of litter at a depth of approximately 6 cm. The NEG treatment was solely provided from day 1, no timing treatment at day 29 was included (as that would mean NEG until day 29 followed by NEG until day 49).
2. The MAT flooring treatment (Figure 1a) consisted of a disinfectant mat (60 × 70 cm mat; product 802010, Agri-Pro Enterprises of Iowa Inc., Iowa Falls, IA, USA) placed in the back middle of the pen under the drinker line. The mats covered 34% of the pen floor surface, and were filled with 3 L of a 1% povidone-iodine solution (diluted with tap water; 050AB Povidone Iodine Solution 10%, Vi-Jon Inc., Breckenridge Hills, MO, USA). Mats were provided on day 1 (MAT-1) or day 29 (MAT-29). Prior to day 29, conditions were identical to the NEG treatment. Every four days, the mats were removed from the pen, rinsed, and refilled with the disinfectant solution. The remainder of the pen contained used litter (66% of floor surface) as in the NEG treatment.
3. The SLAT treatment (Figure 1b) consisted of the mat with the disinfectant solution, placed on top of a black plastic slatted floor (60 × 120 cm, DURA-SLAT^®^ Black Poultry and Kennel Flooring, Southwest Agri-Plastics Inc., Addison, TX, USA). The slatted floor covered 58% of floor surface, but only 24% was accessible to birds as the mat was placed on top of the slatted flooring. The slat and mat were placed on top of the litter but not elevated from the ground. The mat was placed on top of the slatted floor, and both were placed in the back of the pen under the drinker, provided on day 1 (SLAT-1) or day 29 (SLAT-29). The remainder of the pen contained used litter (42% of floor surface) as in the NEG treatment. Prior to day 29, conditions were identical to the NEG treatment. The slatted flooring was removed as needed to eliminate excess litter and fecal content, but was not rinsed. The mat was rinsed and refilled every four days.
4. The POS flooring treatment was provided from day 1 (POS-1) or day 29 (POS-29), with new pine shavings (10.7% moisture content prior to bird placement) at a depth of 6 cm, and shavings completely replaced every four days. Prior to day 29, conditions were identical to the NEG treatment.

Fourteen video cameras (IP Bullet camera FLPB133F, FLIR Systems Inc., Wilsonville, OR, USA) were installed to record behavior in each pen (total of 14 pens). Videos were recorded on Sundays to limit human disturbance during recording. For 2 pens (MAT-1 and MAT-29 treatments) in week 5, the recording from Saturday (no human disturbance during recording) was used instead of the Sunday recording, which was missing.

Behavior was recorded at individual bird-level using scan sampling with a 1 min inter-sampling interval during two 15 min time periods (starting at 10 AM and 6 PM) for all birds in a pen (13 birds per pen), for 4 weeks (week 1, 2, 5, and 6 of age). Birds were not marked for identification, yet the observer was able to ensure all birds were observed based on the bird location at the start of the scan. This resulted in 15 scans per time period (*n* = 195 observations per time period per pen), and a targeted total of 21,840 behavioral entries (13 birds × 15 scans × 4 weeks × 14 pens × 2 time points). These individual behavioral observations were used to calculate pen-level percentages of time spent on each behavior. Due to normal mortality, fewer birds were observed resulting in a total of 21,350 behavioral entries available for analysis. A single observer used Solomon coder version 17.03.22 (Andras Peter, https://solomon.andraspeter.com/ (accessed on 17 May 2019)) to quantify broiler chicken behaviors (Table 1).

Behavioral entries for all birds of the group were converted to percentage (%) of total observations (*n* = 150–195) at pen level within a 15 min time period. Thus, we calculated the percentage of time spent on a specific behavior per pen, per time point, per week. Frequencies were analyzed in JMP^®^ Pro 15.1 (SAS institute Inc., Cary NC, USA). In addition to the analysis of individual behaviors, we grouped behaviors into an “Active” category (Table 1), including all behaviors besides “Passive” (Table 1). The behavioral categories “Out of View” and “Other” (Table 1) were not analyzed, but were included in the total *n* of observations within a specific time period. Visual inspection of data residuals using normal quantile plots showed normal distribution for all but the residuals of “Dustbathing” and “Play”. Normally distributed data were analyzed by age (1, 2, 5, 6 weeks) to account for the decrease in activity (more sitting) as birds age [31]. Analyses with week as fixed factor did show an age effect for all behavioral categories at *p* < 0.05. For these behaviors, mixed models were used for each age category, with treatment (flooring × timing treatment combination; *n* = 7), time (10 AM and 6 PM; *n* = 2) as fixed factors, with pen nested in block as random factors. Dustbathing and play were analyzed with non-parametric Wilcoxon chi-square test, assessing the effect of treatment (flooring × timing treatment combination; *n* = 7) for each sampling week. Post hoc analyses were performed using a nonparametric comparison for all pairs using the Dunn method for joint ranking, which includes a Bonferroni correction.

## 3. Results

**Figure 2:**
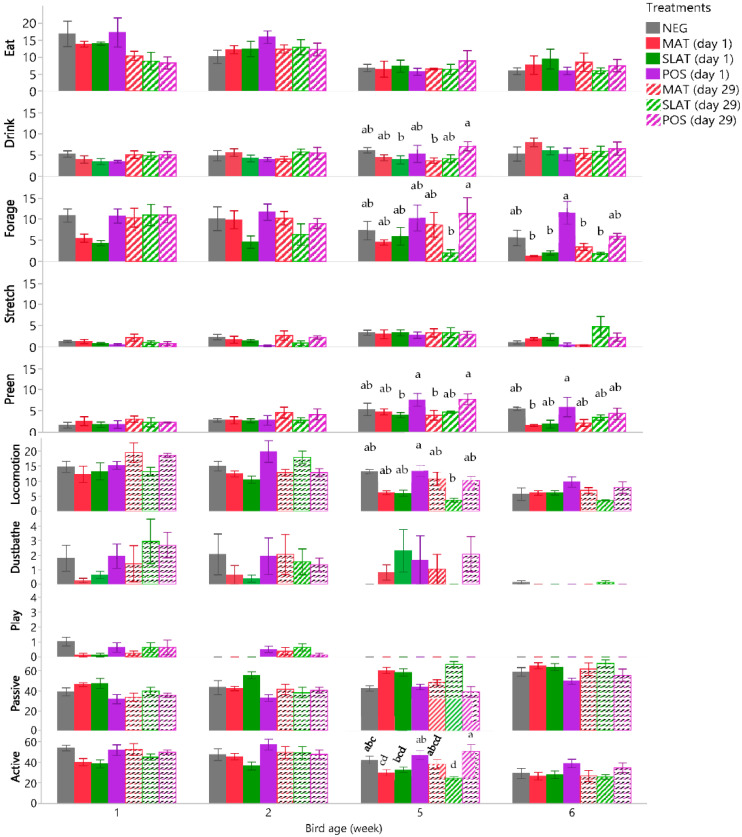
Percentage of observations (mean % ± SEM) spent on eating, drinking, foraging, stretching, preening, locomotion, dustbathing, play, passive, and active behaviors by bird age (in weeks) and by treatments group, with negative control (NEG), iodine mat (MAT), iodine mat with slatted floor (SLAT), and clean litter (POS) provided either from day 1 of age or 29 of age. Within week means without a common superscript (^a–d^) differed at *p* < 0.05.

Effect of flooring treatments on individual behaviors varied during different weeks (Figure 2). In week 1, the percentage of observations spent eating, drinking, foraging, locomoting, preening, stretching, playing and being passive did not differ between the 7 treatment groups (all *p* ≥ 0.13). In week 2, treatments affected the percentage of time spent playing (*p* = 0.047), but pairwise comparisons were non-significant (*p* > 0.45).

In week 5, treatments affected the percentage of time spent drinking, foraging, locomoting, preening, and being active (*p* ≤ 0.035; Figure 2). Percentage of time spent drinking was greater for birds in the POS-29 treatment, compared to the MAT-29 treatment (*p* = 0.025), and the SLAT-1 treatment (*p* = 0.032; Figure 2). Percentage of time spent foraging was greater for broilers in the POS-29 treatment than broilers in the SLAT-29 treatment (*p* = 0.027; Figure 2). Birds in the POS-1 treatment spent more time locomoting than birds in the SLAT-29 treatment (*p* = 0.035; Figure 2). Birds in the POS-1 and POS-29 treatments spent more time preening than birds in the SLAT-1 or MAT-29 treatments (all *p* ≤ 0.042). The sum of all active behaviors in week 5 was greater in the POS-1 treatment than in the SLAT-29 (*p* = 0.005) and MAT-1 treatments (*p* = 0.025). Similarly, the time spent on active behaviors in the POS-29 treatment was greater compared to the SLAT-1 and SLAT-29 treatments, and the MAT-1 treatment (all *p* ≤ 0.01; Figure 2). In week 5, birds in the NEG treatment were more active than birds in the SLAT-29 treatment (*p* = 0.018).

In week 6, treatments affected percentage of time spent foraging (*p* = 0.009) and preening (*p* = 0.022), while other behavior was unaffected (*p* > 0.172). Birds in the POS-1 treatment spent more time foraging compared to birds in all MAT and SLAT treatments (all *p* ≤ 0.030; Figure 2). In week 6, birds in the POS-1 treatment spent more time preening than birds in the MAT-1 treatment (*p* = 0.046; Figure 2).

**Figure 3:**
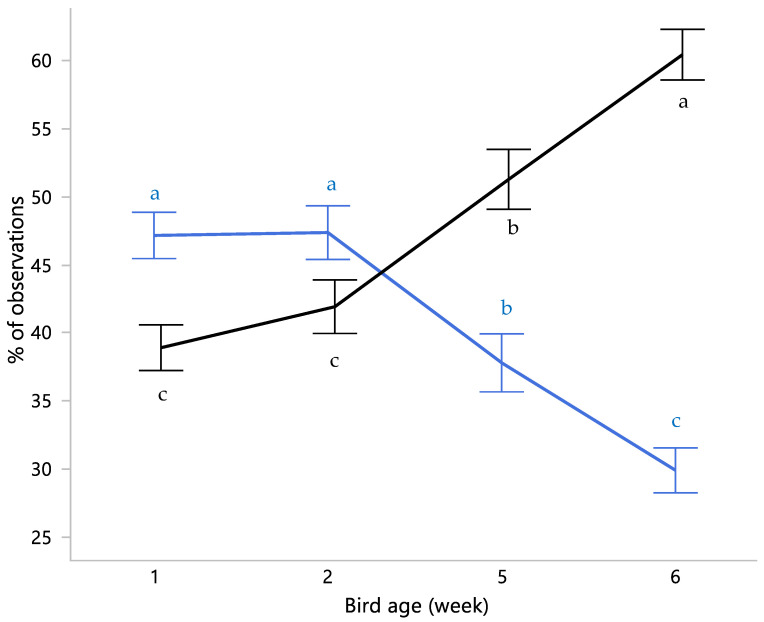
Percentage (means ± SEM) of observations that birds were active (sum of all active behaviors; in blue) or passive (in black) in week 1, 2, 5 and 6. Percentages of active and passive behaviors do not sum to 100% as “out of view” and “other” were excluded from the calculations. Means within a behavioral category without common superscripts differed at *p* < 0.05.

The behavioral repertoire of broiler chickens changed as they aged (all *p* < 0.05; Table 2), with more time spent passive (*p* < 0.001), and less time spent active (*p* < 0.001; Figure 3).

## 4. Discussion

This study evaluated the impact of pen-level flooring treatments on the behavioral repertoire of broiler chickens. In our study this impact was found when birds were 5 and 6 weeks old. Birds housed in the MAT and SLAT treatments generally showed less frequent foraging, preening, locomotion and overall active behaviors than in the POS treatment in week 5 of age, as well as less frequent foraging and preening than in the POS treatment in week 6 of age. Some of these differences in behavioral repertoire could be due to more severe (and possibly painful [23,24]) footpad dermatitis in all MAT and SLAT treatments compared to the NEG and POS treatments. In week 5 and 6, mean footpad dermatitis scores (0–4 categorical scale [32]) were between 1.2 and 2.3 for both MAT treatments and between 1.1 and 2.6 for both SLAT treatments, compared to between 0 and 0.6 for birds in the NEG and POS treatments [25].

Foraging and dustbathing are part of a chicken’s natural behavioral repertoire [2,33]. Birds housed with access to clean, regularly replaced pine wood shavings foraged more than birds housed in some of the MAT and SLAT treatment groups, but similarly to the used litter (NEG) treatment in those weeks. Birds in MAT and SLAT treatment groups may have redirected their foraging behavior towards the feeder because of the limited litter access, as was theorized for broilers housed on a slatted or partially slatted floor [13]. However, time spent eating did not differ between treatments [25]. Access to clean litter promoted foraging at 5 and 6 weeks of age. This result is comparable to findings by [5], who found that clean wood shavings were an attractive foraging substrate. They assessed substrate preferences of commercially housed broilers by providing five different substrates within metal rings. Birds preferred to forage in clean wood shavings compared to straw pellets and the control litter (new shavings from day 1 but not replaced or replenished [5]). However, their birds also preferred to sit inactive in clean shavings compared to some of the other substrates, whilst in our study birds tended to be equally or more active on clean litter. Although not formally analyzed, birds in our MAT and SLAT treatments seemed to prefer spending time in litter rather than on the mat or the slatted flooring, with least behaviors observed on the mat (34% of floor surface; 21% of observations in MAT and 23% in SLAT) or slatted flooring (24% of floor surface; 17% of observations in SLAT) compared to on litter (79% of observations in MAT (66% of floor surface), 60% in SLAT (42% of floor surface); results not presented). This possible aversion to the novel flooring treatments and possible overcrowding in litter could have contributed to the detrimental effect of those treatments on behavior.

As foraging frequency decreased with age in our study and in previous research [5], it is an especially important finding that foraging behavior can be stimulated in older broilers by providing an appropriate substrate. Flooring treatments did not impact the observed prevalence of eating, stretching, dustbathing, play, or general passivity in this study. Prevalences of eating and passivity were comparable as reported for conventionally raised Ross 308 broilers (eat: 10–16% of observations; lie/rest: 52–77% of observations [34]), while dustbathing was observed more frequently in the current study (less than 1% in [34]). Somewhat comparable to our study, enrichments in a commercial setting or in a pilot study also did not impact play behavior in broilers [15,16].

Most active behaviors were observed less frequently with increasing age, including eating, foraging, locomotion, dustbathing, and play. Dustbathing is performed in short periods of time, with peak frequency around midday in laying hens and broilers [31,34]. With observations in our study performed during morning and evening hours, our findings align with expectations and previous research, in that the proportion of time spent dustbathing is small [31,35]. Dustbathing was reduced in week 6 compared to week 1 and 2, but was not significantly impacted by treatments. This suggests that age rather than available substrate affected the ability or motivation to perform dustbathing. As expected, passivity was observed more frequently with increasing age, which is in line with previous research on broilers [5,30,34,35], with 50–60% of observations of fast-growing broilers spent sitting on the floor or resting [31,36]. Drinking increased as birds aged (in line with [35]), whilst stretching and preening peaked at week 5. Early work showed that preening was more frequent and showed different patterns in feed-deprived and thwarted hens compared to hens under normal conditions, suggesting preening as a redirected behavior indicative of frustration [37]. Bokkers and Koene [31] observed broiler behaviors until 12 weeks of age, and found an increase in preening with age. The authors theorized that increased preening may be due to frustration related to poor mobility while birds had an equal motivation to walk compared to at an earlier age. Our results somewhat support the theory that poor mobility at a later age affected the birds’ behavior. Foraging, dustbathing, stretching and locomotion require energetic movements and exercise of the legs [38], and these behaviors were decreased in week 6 compared to week 5 of age. Preening, the behavior potentially indicative of frustration at a later age, was more frequent in week 6 than week 1, but similar to week 2, and less frequent than in week 5. This suggests that as birds age their ability to express certain behaviors is inhibited, likely by their body weight [36,39]. A further investigation of broilers’ ability to perform active behaviors at a later age could focus on the use of analgesia to reduce the pain experience and the hypothesized increase in active behaviors thereafter. In addition, to determine the impact of this inhibited ability, an assessment of affective states could be valuable, for instance using a cognitive bias test [40].

Although treatments did not impact passivity, they did impact active behaviors in week 5, with birds spending most time on active behaviors in the POS-29 treatment. Thus, the novelty of clean litter after 4 weeks of access to used litter stimulated broilers’ activity, although only in week 5 and no longer in week 6. This implies that providing commercially housed broilers with fresh litter later in life could boost their activity even at relatively high body weights, but only for a short period of time.

With two replicates per treatment this study had limited statistical power. Therefore, further research on broiler chickens’ behavioral repertoire and the impact of flooring treatments is recommended. Especially for behaviors that were not impacted by treatments in the current study as low statistical power could lead to type II error (not identifying a significant difference between treatment groups, thus a false negative).

## 5. Conclusions

Our results indicate that access to partially slatted flooring and/or disinfectant mats does not benefit broiler chicken welfare in terms of their ability to express highly motivated behaviors. We identified a detrimental effect of these novel flooring types (slats and/or mats) on the expression of foraging, preening, locomotion and overall active behaviors in week 5 of age, as well on foraging and preening in week 6 of age. Thus, our results suggest that access to clean, regularly replaced litter, is beneficial for broiler chicken welfare in terms of their ability to express their normal behavioral repertoire.

## Figures and Tables

**Table 1:** Ethogram of recorded broiler chicken behaviors.

Behavior	Description
Eat	Beak inside or above feeder, may include extension of the neck
Drink	Beak near or in contact with the drinker, may include extension of the neck
Forage ^1^	Pecking/scratching at the flooring substrate
Stretch	Extension of the wing or leg, may include fluffing of the feathers
Preen ^2^	Feathers are raised, cleaned and realigned with the beak
Locomotion ^3^	Moving using legs in a continuous forward motion (walking or running)
Dustbathe ^1^	Vertical wing shakes, interacting with flooring substrate, performing side-rubs, and intermittent ground pecking with beak
Play ^4^	Spontaneous motor behavior that occurs without apparent purpose. Includes frolicking (sudden running with no apparent stimulus, flapping wings) and food running (object in beak and locomotion at high speed)
Passive ^1,3^	Bird sits resting its abdomen on the flooring substrate or stands with feet in contact with any flooring. Bird may have head tucked under the wing or have head at or below body level. Bird may stand without showing other behaviors.
Other	Other behaviors or behavior cannot be identified
Out of View	Bird is out of camera view
Active	Sum of all active behaviors, including eat, drink, forage, stretch, preen, locomotion, dustbathe, and play

^1^ Adapted from [28]. ^2^ [29]. ^3^ Adapted from [30]. ^4^ Adapted from [16].

**Table 2:** Behaviors as percentage of observations at pen-level (mean % ± SE) during week 1, 2, 5 and 6 of life.

Behavior	Week				Age Effect (*p*-Value)
	1	2	5	6	
Eat	12.80 ± 1.07 ^a^	12.61 ± 0.69 ^a^	6.88 ± 0.63 ^b^	7.29 ± 0.73 ^b^	<0.001
Drink	4.47 ± 0.29 ^b^	4.87 ± 0.34 ^ab^	4.97 ± 0.43 ^ab^	6.05 ± 0.47 ^a^	0.034
Forage	9.14 ± 0.78 ^a^	8.85 ± 0.81 ^a^	7.17 ± 1.00 ^a^	4.56 ± 0.77 ^b^	<0.001
Stretch	1.12 ± 0.18 ^b^	1.64 ± 0.26 ^b^	3.15 ± 0.30 ^a^	1.86 ± 0.45 ^b^	<0.001
Preen	2.22 ± 0.28 ^c^	3.26 ± 0.34 ^bc^	5.47 ± 0.47 ^a^	3.57 ± 0.49 ^b^	<0.001
Locomotion	15.26 ± 0.89 ^a^	14.49 ± 0.87 ^a^	9.06 ± 0.81 ^b^	6.57 ± 0.56 ^c^	<0.001
Dustbathe ^1^	1.67 ± 0.36 ^a^	1.42 ± 0.35 ^a^	1.13 ± 0.39 ^ab^	0.04 ± 0.03 ^b^	<0.001
Play ^1^	0.50 ± 0.11 ^a^	0.24 ± 0.07 ^ab^	0.00 ± 0.00 ^c^	0.00 ± 0.00 ^c^	<0.001
Passive	38.92 ± 1.68 ^c^	41.93 ± 1.96 ^c^	51.28 ± 2.20 ^b^	60.43 ± 1.86 ^a^	<0.001
Active	47.16 ± 1.70	47.37 ± 1.97	37.81 ± 2.13	29.92 ± 1.65	<0.001
Other	4.56 ± 0.35	4.06 ± 0.28	2.96 ± 0.24	2.64 ± 0.37	-
Out of view	9.36 ± 0.78	6.63 ± 0.73	7.95 ± 0.70	7.01 ± 0.69	-

Means within a row without a common superscript (^a–c^) differed at *p* < 0.05; ^1^ Non-parametric analysis; - Not assessed.

## Data Availability

The data presented in this study are available upon request from the corresponding author.
